# GSTO2 Isoforms Participate in the Oxidative Regulation of the Plasmalemma in Eutherian Spermatozoa during Capacitation

**DOI:** 10.3390/antiox8120601

**Published:** 2019-11-29

**Authors:** Lauren E. Hamilton, Michal Zigo, Jiude Mao, Wei Xu, Peter Sutovsky, Cristian O’Flaherty, Richard Oko

**Affiliations:** 1 Department of Biomedical and Molecular Sciences, Queen’s University, Kingston, ON K7L 3N6, Canada; 9leh5@queensu.ca (L.E.H.); wx@queensu.ca (W.X.); 2 Division of Animal Sciences, College of Food, Agriculture and Natural Resources, Columbia, MO 65211, USA; zigom@missouri.edu (M.Z.); maoj@missouri.edu (J.M.); sutovskyp@missouri.edu (P.S.); 3 Division of Obstetrics, Gynecology and Women’s Health, School of Medicine, University of Missouri, Columbia, MO 65211, USA; 4 Department of Surgery (Urology Division), Faculty of Medicine, McGill University, Montreal, QC H4A 3JI, Canada; cristian.oflaherty@mcgill.ca

**Keywords:** glutathione-s-transferase omega 2, capacitation, fertilization, male fertility, oxidative regulation, spermatozoa, reactive oxygen species (ROS)

## Abstract

In addition to perinuclear theca anchored glutathione-s-transferase omega 2 (GSTO2), whose function is to participate in sperm nuclear decondensation during fertilization (Biol Reprod. 2019, 101:368–376), we herein provide evidence that GSTO2 is acquired on the sperm plasmalemma during epididymal maturation. This novel membrane localization was reinforced by the isolation and identification of biotin-conjugated surface proteins from ejaculated and capacitated boar and mouse spermatozoa, prompting us to hypothesize that GSTO2 has an oxidative/reductive role in regulating sperm function during capacitation. Utilizing an inhibitor specific to the active site of GSTO2 in spermatozoa, inhibition of this enzyme led to a decrease in tyrosine phosphorylation late in the capacitation process, followed by an expected decrease in acrosome exocytosis and motility. These changes were accompanied by an increase in reactive oxygen species (ROS) levels and membrane lipid peroxidation and culminated in a significant decrease in the percentage of oocytes successfully penetrated by sperm during in vitro fertilization. We conclude that GSTO2 participates in the regulation of sperm function during capacitation, most likely through protection against oxidative stress on the sperm surface.

## 1. Introduction

The spermatozoa expelled from the seminiferous tubules at the end of spermatogenesis lack progressive motility and the ability to fertilize the oocyte. It is only through epididymal maturation and functional capacitation that spermatozoa undergo the necessary transformational changes needed to fertilize. 

Capacitation is a highly orchestrated set of reactions that ultimately culminates in the acrosome exocytosis reaction and spermatozoa obtaining their fertilizing competency as they approach the oocyte in the oviduct [1]. Due to the lack of transcriptional and translational activity within mature spermatozoa, any changes that the cell undergoes must occur through post-translational modifications of pre-existing proteins. A key modulator of cell signaling and enzymatic function are the levels of reactive oxygen species (ROS) [2,3,4,5,6,7,8].

High sensitivity to membrane lipid peroxidation requires eutherian spermatozoa to maintain a fine balance between the production and regulation of ROS [9,10,11,12]. High concentrations of oxygen radicals and peroxidation by-products have been well documented as contributors to male infertility [9,13,14,15,16,17]. Ideally, when ROS is maintained at low concentrations, the ROS molecules are utilized by spermatozoa to modulate cellular functions such as the initiation of capacitation and hyperactivated motility [1,7,18,19,20,21,22]. Spermatozoa are, therefore, critically dependent on their network of regulatory and detoxifying enzymes to ensure that optimal concentrations of ROS are maintained.

Eutherian spermatozoa are largely devoid of the cytoplasmic reservoir of antioxidant enzymes seen in most somatic cells, and therefore have a large reliance on the detoxifying capacity of the fluids in their surroundings. Epididymal secretions and semen are amongst the most antioxidant rich fluids within the body, equipped with specialized ROS scavenging molecules and enzymes that aid in buffering the oxidative stress levels of sperm cells [23,24,25,26,27]. It is also through these secretions that surface-anchored detoxifying enzymes can be imparted to the plasmalemma of spermatozoa as they progress through the male reproductive system. 

The proteomic analysis of epididymal secretions, semen and surface bound spermatozoon proteins helps to decode the detoxifying landscape of spermatozoa and identify the regulatory systems at play [28,29]. At the center of many detoxifying systems is the tripeptide thiol glutathione. Glutathione provides a recyclable source of reducing power and facilitates many groups of antioxidant enzymes residing within and on the sperm surface, such as glutathione reductases, glutaredoxins, thioredoxins, perioxiredoxins and glutathione-s-transferases. 

Glutathione-s-transferases (GSTs) are a large super-family of phase II detoxification enzymes that are ubiquitously found throughout the body, and well represented within the male reproductive environment [30]. GSTs of the Mu, Pi, Theta, Alpha, Zeta and Omega classes have all been identified as components of the seminal plasma or as sperm resident proteins, with their own functionally distinct roles [31,32,33,34]. Furthermore, several classes of GST have recently been shown to have functional multimodality, acting in both sperm–egg interactions and redox regulation of the plasmalemma [31]. Harboring enzymes that can facilitate more than one cellular process may be a valuable asset for cells such as spermatozoa that have evolved to be streamlined and devoid of most cytosolic resources.

The Omega class of GSTs have also been shown to have multifunctionality [30,35,36]. Equipped with a cysteine residue at their activity site, GSTOs are not only able to facilitate glutathione reductase reactions but have also been shown to have dehydroascorbate reductase capabilities [30,35,36]. Moreover, GSTO2, one of only two functionally active enzymes within the Omega class, has been credited as having the highest levels of dehydroascorbate reductase functionality within mammals [37]. With the epididymis and seminal plasma having some of the highest concentrations of ascorbic acid (AA) found within the body, maintaining optimal concentrations of AA within the sperm may prove vital to its fitness [26]. Therefore, the enzymatic versatility of facilitating reactions in both glutathione and AA centered pathways may make GSTO2 a highly valuable surface-bound enzyme.

In addition to its previous characterization as a constituent of the postacrosomal sheath and perforatorium of the perinuclear theca [34,38], we demonstrate that GSTO2 is also present on the sperm surface in mouse and boar spermatozoa. Through surface protein isolation, indirect immunofluorescence, fluorescence immunohistochemistry, functional inhibition studies and computer assisted sperm analysis, we characterize the surface localization of GSTO2 isoforms and demonstrate the functional role of GSTO2 in facilitating sperm capacitation through oxidative regulation.

## 2. Materials and Methods

### 2.1. Animals

Retired breeder CD1 and C57BL/6 mice were purchased from Charles River Laboratories (Charles River, St-Constant, QC, Canada). All procedures in this study were conducted under the Animal Utilization Protocols approved by Queen’s University Animal Care Committee (protocol # 2017-1742) and complied with the Guidelines of the Canadian Council on Animal Care. Fertile, non-transgenic boar semen samples were collected at the University of Missouri’s National Swine Resource and Research Center and processed in their Division of Animal Sciences, College of Food, Agriculture and Natural Resources (Columbia, MO, USA), under the strict guidance of the University of Missouri’s Animal Welfare Assurance Number and Animal Care and Use Committee (ACUC) protocol # A3394-01.

### 2.2. Sperm Extractions from Mice

Spermatozoa were obtained from the fresh cauda epididymis’ of mature CD1 and C57BL/6 males. Cauda epididymis’ were placed in approximately 0.5 mL of phosphate-buffered saline (PBS) and pierced with a 26 ½ gauge needle to allow the sperm to diffuse out into the solution.

### 2.3. Antibodies and Reagents

The central antibody was a goat polyclonal anti-GSTO2 antibody (Y-12, Santa Cruz Biotechnology, Dallas, TX, USA), used at a concentration of 0.2 µg/mL for Western blot analysis, and 6.67 µg/mL for fluorescence immunocytochemistry. For the measure of protein tyrosine phosphorylation, the clone 4G10 anti-tyrosine phosphorylation antibody (Millipore-Sigma, 05-321, St. Louis, MO, USA) was used at a concentration of 0.1 µg/mL and standardized using an anti-alpha tubulin antibody (Sigma T6074, Burlington, MA, USA). For immunohistochemistry a rabbit-polyclonal anti-GSTO2 (Sigma Prestige, HPA048141, Burlington, MA, USA) was used at a concentration of 6.67 µg/mL. For Western blot analysis, a rabbit anti-goat IgG-HRP (horseradish peroxidase) (0.4 µg/mL, Santa Cruz Biotechnology, Santa Cruz, CA, USA) secondary antibody was used, and, for indirect immunofluorescence studies, a donkey anti-goat IgG-CFL (colorized fluorochrome) 555 (Santa Cruz, 2 µg/mL), or donkey-anti-rabbit-IgG-CFL 488 was used (2 µg/mL, Abcam, Cambridge, MA, USA). For the assessment of the acrosome exocytosis reaction lectin PNA (*Arachis hypogaea)* conjugated to the colorized fluorocrome 647 (Invitrogen, L32460, Waltham, MA, USA) was used at a concentration of 15 µg/mL. For peroxidation analysis, a BODIPY 581/591 C11 probe (4,4-difluoro-5-(4-phenyl-1,3-butadienyl)-4-bora-3a,4a-diaza-s-indacene-3-undecanoic acid) (Invitrogen Molecular Probes, D3861) was used at a final concentration of 5 µM. For evaluation of the total reactive oxygen species, the Cellular ROS Detection Assay kit (ab186029Abcam, Cambridge, MA, USA) was used following the manufacturer’s manual. For all enzymatic inhibition studies, a membrane permeable cell tracker probe (Invitrogen Molecular Probes C7025) was used. The probe has been shown to bind covalently and irreversibly to the active site of GSTO isozymes [39] and was used at a concentration of 100 µM in all our experiments. Any additional reagents were purchased from Millipore-Sigma (Burlington, MA, USA).

### 2.4. Fluorescence Electrophoresis and Western Blotting Analysis

All sperm samples were freshly extracted and subsequently incubated with our inhibitory probe for 25 min at 37 or 38 °C, depending on the species. The samples were washed twice and then solubilized in a non-reducing sample buffer (200 mM Tris pH 6.8, 4% SDS, 0.1% bromophenol blue, 40% glycerol, 5% β-mercaptoethanol). A BLUeye pre-stained protein ladder (GeneDirex) was loaded along with approximately 1–2 million cells per lane and resolved on 4% stacking and 12% separating polyacrylamide gels, as described by Laemmli [40]. The gel was run at 100 volts for 110 min before being placed in transfer buffer and imaged in a fluorescence biophotonic chamber. After transfer to a polyvinylidene fluoride (PVDF) membrane (Millipore) for 120 min in Tris-glycine transfer buffer on ice using a Hoefer Transfer apparatus (Hoefer Scientific Instruments), the membrane was also imaged in the same fluorescence chamber. The membrane was then blocked in a 10% skim milk and phosphate-buffered saline (PBS) solution with 0.05% Tween-20 (PBS-T) for 30 min to prevent non-specific binding. The membrane was incubated with primary antibody overnight at 4 °C with slight agitation. The next day, the membrane was washed in PBS-T six times, each for five minutes, before a two hour incubation with secondary antibody conjugated to horseradish peroxidase. The membrane was then washed extensively and subjected to an immunodetection reaction that was visualized using Clarity Western ECL Substrate (Bio Rad Laboratories, Hercules, CA, USA). The membrane was exposed to X-ray film for developing. For the evaluation of tyrosine phosphorylation (PY) during capacitation, relative intensities were calculate using Image J. Total PY was calculated for each sample and normalized using the intensity of tubulin in each sample.

### 2.5. Fluorescence Immunocytochemistry 

Mouse and boar spermatozoa were mounted on poly-L lysine coated coverslips and fixed in 2% formaldehyde for 40 min. Non-specific binding was blocked using 5% bovine serum albumin (BSA) for 25 min before incubation in primary antibody overnight at 4 °C. The next day, the coverslips were washed extensively in 1% BSA-PBS before a 40 min incubation with secondary antibody conjugated to a fluorescent marker and DAPI (4’,6-Diamidino-2-Phenylindole, Dihydrochloride) at room temperature and hidden from light. The coverslips were washed again before being mounted on glass slides using VectaShield mounting medium (Vector Laboratories, Burlingame, CA, USA) and sealed with nail polish. Spermatozoa from mouse and boar were also incubated with our fluorescent inhibitor for 25 min, washed extensively and mounted onto glass slides to visualize the binding pattern. Fluorescence images were taken at the Queen’s University Cancer Research Institute Imaging Centre, using a Quorum Wave Effects spinning disc confocal microscope or at the University of Missouri-Columbia, using a Nikon Eclipse 800 microscope with CoolSnap CCD camera (Nikon, Tokyo, Japan). All images were subsequently analyzed using MetaMorph Imaging Software (Molecular Devices, San Jose, CA, USA).

### 2.6. Boar Surface Protein Extractions

Boar surface sperm protein extractions were performed on approximately 3 × 10^8^ cells of both ejaculated and in vitro capacitated samples, as previously described in [41] with the Pierce Surface Protein Biotinylation kit (ThermoFisher Scientific, A44390). Extracts were subsequently precipitated with the use of a 2-D Clean up Kit (GE Healthcare, Uppsala, Sweden) and resolubilized with Laemmli reducing sample buffer for Western Blot Analysis.

### 2.7. Fluorescent Immunohistochemistry

Paraformaldehyde fixed, and paraffin embedded boar and mouse epididymal sections were deparaffinized in xylene and hydrated through a graded series of methanol solutions. After hydration, the sections were treated to abolish autofluorescence and subjected to antigen retrieval by microwave irradiation in a 5% Urea, Tris-HCL solution, pH 9.5 [18]. Before primary antibody incubation, sections were blocked with 5% normal goat serum (NGS) diluted with phosphate-buffered saline (PBS). The primary antibody was made in 1% NGS-PBS and incubated at 4 °C overnight. Slides were washed with 1% NGS-PBS before incubation with fluorescent-tagged secondary antibody and DAPI. Once completed, slides were washed in PBS before being mounted to coverslips using Vectashield mounting Media (Vector Laboratories) and sealed with clear nail polish. Images were captured at the Queen’s University Cancer Research Institute Imaging Centre, using a Quorum Wave Effects spinning disc confocal microscope and analyzed using MetaMorph imaging software.

### 2.8. Mouse In Vitro Capacitation and Acrosome Exocytosis Reaction

Spermatozoa were extracted from fresh cauda epididymis by piercing with a 26 ½ gauge needle and allowing the sperm to diffuse out into a Whittens-Hepes Medium. Sperm were then incubated for 25 min at 37 °C, 5% CO_2_ in Whittens-Hepes Medium with either the inhibitor, or dimethyl sulfoxide (DMSO, Control). After treatment, sperm were diluted to a final concentration of approximately two million cells/mL with either a capacitation medium (Modified Whittens-Hepes medium supplemented with 5mg/mL BSA and 20 mM NaHCO_3_) or the non-capacitiation medium (Whittens-Hepes without supplementation) and left to incubate for up to 90 min at 37 °C, 5% CO_2_. For the analysis of tyrosine phosphorylation, samples were collected every 45 min, washed twice and placed in sample buffer before being run on a Western blot. For the acrosome exocytosis reaction, sperm cells were left to incubate in either capacitating or non-capacitating media for 60 min and then progesterone was added to a final concentration of 10 µM and left for an additional 30 min. Cells were subsequently washed in PBS and allowed to air dry on glass slides. Cells were fixed with ice cold absolute methanol for 15 min, washed in PBS and stained with PNA (15µg/mL) and DAPI for 30 min. Slides were rinsed one final time and coverslips were mounted. In each treatment, 200 cells were randomly selected and the acrosome was scored as either intact or reacting/reacted. At least three replicates of each treatment were assessed and the reported values represent the average per group. The Western blotting results were analyzed using an ANOVA with a post-hoc Tukey test, comparing the mean intensities of three replicates. The acrosome exocytosis reaction results were compared using a t-test with Welch’s correction, comparing the average percentage of reacted cells from three replicates.

### 2.9. Boar In Vitro Capacitation

Boar spermatozoa were collected from fresh ejaculate and were subsequently centrifuged and washed to remove seminal plasma. Sperm were counted using a hemocytometer (ThermoFisher Scientific, Waltham, MA), and incubated in a modified TL-HEPES medium with either the GSTO Inhibitor or DMSO (Control) for 25 min at 38 °C. Sperm cells were then placed in either capacitating (modified TL-HEPES supplemented with 5 mM sodium pyruvate, 11 mM D-glucose, 2 mM CaCl_2_, 2 mM sodium bicarbonate, and 2% (*m/v*) bovine serum albumin) or non-capacitating medium (modified TL-HEPES medium without supplementation) to a concentration of two million cells/mL and incubated at 38 ° C for four hours. Cells were removed every hour, washed, and placed in reducing sample buffer, for tyrosine phosphorylation analysis by Western blotting up until the four-hour time point. The results were analyzed using an ANOVA with a post-hoc Tukey test, comparing the mean intensities from three replicates.

### 2.10. Mouse In Vitro Fertilization

Mouse oocytes were obtained from super-ovulated 6 week old CD1 females. Females were given 10 UI of pregnant mare serum gonadotropin (PMSG) through intraperitoneal (IP) injection, followed 48 h later by 10 UI of human chorionic gonadotropin (hCG), also administered through IP injection. Oviducts were harvested from the sacrificed females 12–13 h after the last hCG injection into Advanced KSOM medium (MR-101-D, Millipore-Sigma), warmed to 37 °C. Cumulus-oocyte complexes were extracted from the oviducts into warmed Advanced KSOM medium, washed and rested at 37 °C, 5% CO_2_ under mineral oil until fertilization droplets were prepared, to a maximum of 30 min. Spermatozoa were extracted from the cauda epididymis and into Whitten-Hepes Medium where they were either incubated with the GSTO inhibitor (100 µM) or DMSO for 25 min under mineral oil at 37 °C, 5% CO_2_. Sperm were subsequently washed and placed into EmbryoMax human tubal fluid (HTF, MR-070-D, Millipore-Sigma) to capacitate for a minimum of 1 h. Sperm were then diluted with HTF into a 50 µl droplet with a concentration of approximately 1 × 10^5^/mL sperm and between 20–25 oocytes were added to each droplet. Fertilization droplets were incubated at 37 °C, 5% CO_2_ under mineral oil for 5 h before oocytes were removed, washed and further cultured in 50 µL Advanced KSOM droplets under mineral oil for approximately 8 h. Oocytes were then fixed in 2% formaldehyde, permeabilized in phosphate-buffered saline with 0.1% Triton-X-100 (PBS-Tx) and stained with DAPI to allow for the visualization of the sperm head or pronuclear formation, indicative of successful sperm penetration. Oocytes were then mounted onto glass slides using Vectashield mounting Medium (Vector Laboratories) and sealed with clear nail polish. Images were captured at the Queen’s University Cancer Research Institute Imaging Centre, using a Quorum Wave Effects spinning disc confocal microscope. Three replicates of each treatment were performed, all using different mice, and statistical analysis was performed using a t-test with Welch’s correction. 

### 2.11. Swine In Vitro Fertilization

Ovaries were obtained from a local slaughterhouse and aspirated to obtain oocytes from follicles of 3–6 mm in size. Oocyte with uniform ooplasm and compact cumulus cells were selected and in vitro matured at 38.5 °C, 5% CO_2,_ for 42 to 44 h. Cumulus cells from matured cumulus-oocyte complexes (COCs) were removed with 0.1% hyaluronidase in TL-HEPES-PVA and washed three times with TL-HEPES-PVA medium. Twenty-five to thirty oocytes were placed into 100 µL drops of the mTBM medium, while sperm were prepared. Boar semen was collected from sperm rich fraction the day before IVF. Sperm cells were incubated with the GSTO inhibitor for 25 min at 38.5 °C immediately after being isolated from the semen and before being placed in a short BTS extender (BTS, IMV Technologies, Maple Grove, MN, USA) until used. Mitochondria of the sperm tail were stained with a viable, mitochondrion-specific probe MitoTracker^®^ Red CMXRos (Molecular Probes, Inc., Eugene, OR, USA) for 10 min at 38 °C in a warm incubator before the sperm solution was diluted to a concentration of 1 × 10^6^ cells/mL. Co-incubation of the sperm and the oocytes was left for approximately 6 h before oocytes were removed and transferred to 100 µL drops of PZM-3 medium containing 0.4% BSA (A6003; Sigma, Burlington, MA, USA) for additional culture. Three replicates of each experiment were performed using three different ejaculates and presented as the average. Statistical analysis was performed using a t-test with Welch’s correction.

### 2.12. Mouse Computer-Aided Sperm Analysis (CASA)

Spermatozoa were extracted from the cauda epididymis of mature C57BL/6 males and placed in Whittens-Hepes Medium. Sperm were subsequently placed with the GSTO inhibitor (100 μM) or DMSO (Control) for 25 min at 37 °C before being washed and diluted with additional Whittens-Hepes medium or capacitating medium (Whittens Hepes supplemented with 5mg/mL BSA and 20 mM NaHCO_3_) to a final concentration of approximately 1–2 million cells/mL. Sperm were then placed back at 37 °C and left to capacitate for one hour. Samples were then gently spun down and resuspended for analysis. Analysis was done by Sperm Vision HR software version 1.01 (Minitube, Ingersoll, ON, Canada). At least 200 cells from each treatment were analyzed in each experimental trial. All cells that were not unequivocally identifiable as sperm were removed from the analysis by the technician. Four replicates were performed, each using a new mouse, and the data are presented as the average of all replicates. Multiple t-tests were performed to determine statistical significance.

### 2.13. Lipid peroxidation Intensity Analysis

Mouse spermatozoa were extracted from the cauda epididymis of mature C57BL/6 males and placed in Whitten-Hepes Medium. Sperm were diluted to a concentration of approximately 2 × 10^6^/mL, and subsequently placed with the BODIPY 581/591 C11 (D3861, Invitrogen Molecular Probes, Eugene, OR, USA) to a final concentration of 5 µM for 20 min at 37 °C. A subset of sperm were not treated with the BODIPY C11 probe to act as a baseline for innate fluorescence. Sperm were washed twice at 650× *g* for 5 min before being placed with the GSTO inhibitor (100 µM) or DMSO (Control) for 25 min at 37 °C. Spermatozoa were washed again and placed in capacitating medium (Whittens-Hepes Medium supplemented with 5mg/mL BSA and 20 mM NaHCO_3_) to a final concentration of approximately 1–2 million cells/mL and incubated for one hour at 37 °C, with agitation every 15 min. Spermatozoa were subsequently washed and placed on a glass slide, covered with a coverslip and sealed with clear nail polish. Three trials were performed for each treatment, and at least 200 cells per treatment were analyzed each trial by confocal microscopy. Images were captured at the Queen’s University Cancer Research Institute Imaging Centre, using a Quorum Wave Effects spinning disc confocal microscope, and analysis was performed using the MetaMorph Imaging software. The intensities of both the red and green fluorescence were acquired for all treatments and controls. For GSTO Inhibited samples, the fluorescent intensity of the inhibitor itself was subtracted from the overall green intensity to ensure it did not impact the overall ratio of green and red fluorescence. The results were analyzed using a t-test with Welch’s correction to determine statistical significance.

### 2.14. Cellular Reactive Oxygen Species Levels

Mouse spermatozoa were extracted from the cauda epididymis of mature CD1 males and placed in Whitten-Hepes Medium. The sperm were subsequently placed with the GSTO inhibitor (100 µM) or vehicle (DMSO) for 25 min at 37 °C. Spermatozoa were then diluted to a concentration 5 × 10^6^ cells/mL and placed under capacitating conditions through supplementing the buffer with 5 mg/mL BSA and 20 mM NaHCO_3_. Spermatozoa were left to incubate for 60 min, with agitation every 15 min at 37 °C/5% CO_2_. Reactive oxygen species levels were then probed using the Cellular ROS Assay Kit (ab186029). Samples were subsequently washed and fixed in 2% formaldehyde before being analyzed using flow cytometry. Flow cytometry was performed at the Queen’s University Cardiac Pulmonary Unit using the Sony SH800 Cell Sorter and least 10,000 events were recorded for each sample. Three experimental trails were done for each treatment, each using a different mouse, and the data presented include the average mean intensity over the three trials. Statistical analysis was done using a t-test with Welch’s correction.

### 2.15. Statistical Analysis

Statistical analysis was performed using Prism 8 statistical software. An ANOVA with a Tukey post-hoc test was used for the statistical evaluation of the results obtained from in vitro capacitation experiments. Multiple t-tests were performed for the computer-aided sperm analysis and acrosome exocytosis reaction experiments comparing different treatment groups under the same testing conditions. A t-test with Welch’s correction was performed in the lipid peroxidation, total cellular ROS levels and in vitro fertilization experiments. All experiments were performed at least three times and all results are the averages of all replicates. For each experiment, all replicates of mouse experiments were performed with a different mouse, whereas all experiments performed in boar used a different ejaculate.

## 3. Results

### 3.1. The presence of GSTO2 on the Plasmalemma of Mature Mouse and Boar Spermatozoa

**Figure 1:**
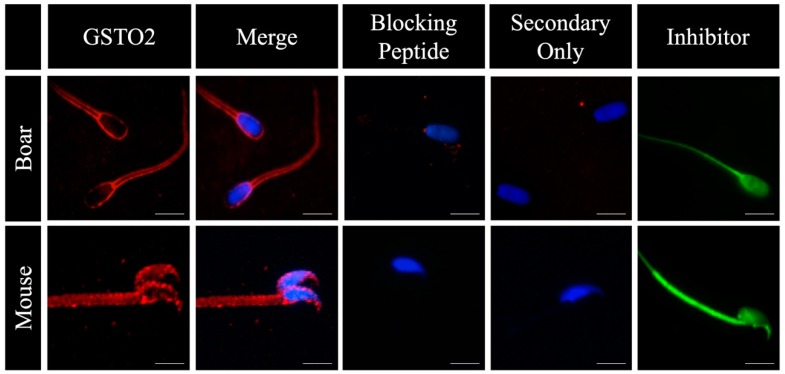
The surface reactivity of glutathione-s-transferase omega 2 (GSTO2) on non-permeabilized mouse and boar spermatozoa using indirect immunofluorescence. Non-permeabilized fresh boar and mouse spermatozoa were stained using a GSTO2 specific (Y-12) antibody (GSTO2), or a GSTO-specific fluorescent inhibitor (Inhibitor). Nuclei were labelled with DAPI. To confirm the specificity of the antibody, the anti-GSTO2 antibody was preincubated with its blocking agent (blocking peptide, the peptide used to raise the antibody) according to the manufacturer’s instructions and a secondary-only control was done (Secondary Only). The GSTO inhibitor is a membrane permeable cell tracker probe (Invitrogen Molecular Probe C7025). The bar represents 10 μm.

Glutathione-S-Transferase Omega 2 was observed on the plasmalemma of non-permeabilized mouse and boar spermatozoa through the use of indirect immunofluorescence (Figure 1). This reactivity was inhibited when the antibody (Y-12) was pre-incubated with its corresponding peptide block, and was absent when samples were incubated in only the secondary antibody. Furthermore, when spermatozoa were incubated with a Glutathione-S-Transferase Omega (GSTO)-specific inhibitory binding molecule, the entirety of the sperm showed reactivity, suggesting enzymes of the GSTO family are present outside of the perinuclear theca, the sole localization of GSTO enzymes currently documented within eutherian spermatozoa [34].

**Figure 2:**
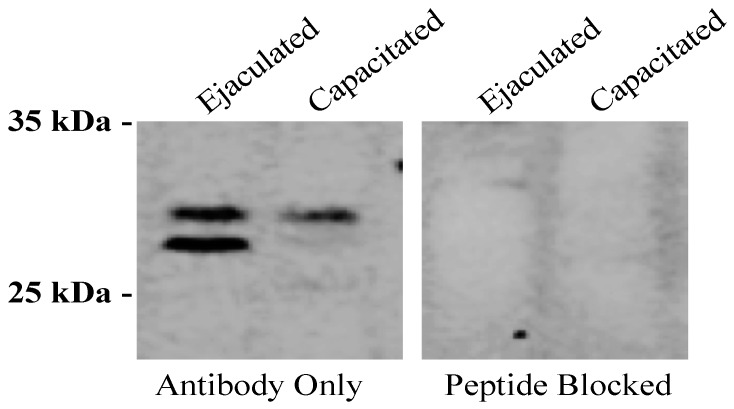
GSTO2 reactivity to biotin-isolated surface proteins of ejaculated and capacitated boar spermatozoa. Immunoblotting using an anti-GSTO2 (Y-12) antibody on biotin-isolated surface proteins from ejaculated boar sperm (Lane 1) and capacitated boar sperm (Lane 2). The antibody was pre-incubated with its corresponding blocking peptide to show specificity in both the ejaculated boar sperm (Lane 3) and capacitated boar sperm (Lane 4) samples.

To further investigate the surface localization of GSTO2, surface proteins of non-permeabilized fresh and capacitated boar spermatozoa were isolated through the use of the Pierce Cell Surface Biotinylation and Isolation Kit (A44390, ThermoFisher Scientific, Waltham, MA, USA), run on an SDS-PAGE gel and probed with an anti-GSTO2 antibody. The findings revealed that two GSTO2 isoforms were present on the surface of fresh boar spermatozoa (Figure 2, Lane 1) but that, following in vitro capacitation, only the higher isoform remained (Figure 2, Lane 2). This reactivity was inhibited when the antibody was pre-incubated with its corresponding peptide block (Figure 2, Lanes 3 and 4).

**Figure 3:**
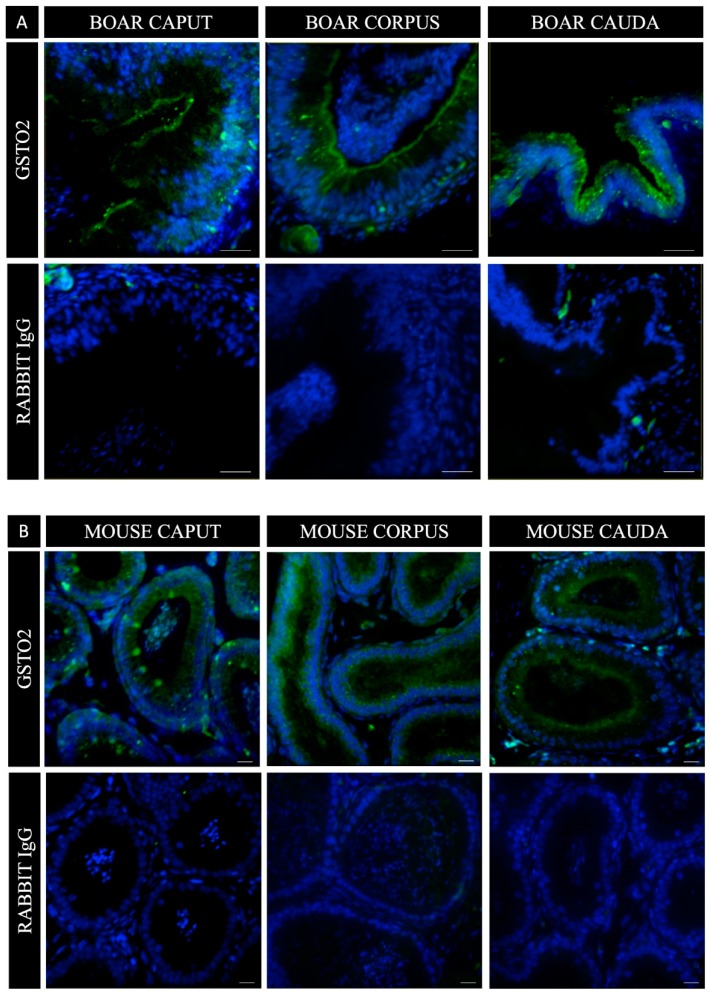
GSTO2 reactivity in histological sections of boar (**A**) and mouse (**B**) epididymis. In both species, caput, corpus and cauda epididymal sections show heightened GSTO2 reactivity at the luminal aspect of the epididymal epithelium with some fissures of reactivity towards the basal membrane. In mouse caput epididymal sections, some isolated pockets of reactivity can also be seen, spanning the length of the epididymal epithelium. Nuclear material was stained with DAPI. The bars in both (**A**) and (**B**) is representative of 20 μm.

Due to the spermatozoon’s lack of transcriptional and translational capabilities after the round spermatid stage of spermiogenesis and the absence of GSTO2 membrane reactivity previously reported during spermatogenesis [34], we aimed to investigate if there was an external source of the enzyme that was secreted during epididymal transport. Fluorescence immunohistochemical staining of porcine and murine epididymal sections (Figure 3) revealed the presence of GSTO2 within the caput, corpus and caudal regions of the epididymis. The reactivity was concentrated at the luminal aspect of the epithelium, however, in the mouse caput, the GSTO2 reactivity was also seen in distinct regions, that spanned from the basal to the luminal aspect of the epithelium.

### 3.2. The Functional Significance of GSTO2 During Capacitation

Many surface proteins secreted by the epididymis have been shown to function in the regulation of capacitation. Therefore, through functional inhibition, using a specific inhibitor that binds to the active site of GSTO enzymes (Figure S1), we sought to determine if GSTO2 has a role modulating some facet of the capacitation process. Inhibitor specificity was confirmed in both mouse and boar whole sperm through fluorescence gel electrophoresis (Figure S2).

**Figure 4:**
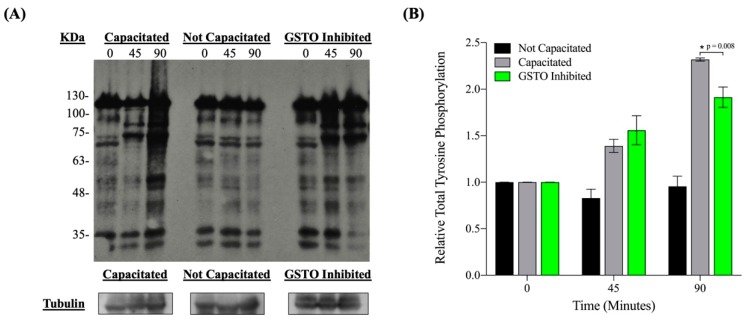
The level of protein tyrosine phosphorylation during in vitro capacitation in mouse spermatozoa. (**A**) Total protein tyrosine phosphorylation patterns at 0, 45 and 90 min after in vitro capacitation in DMSO-treated and capacitated (Capacitated), DMSO-treated and not capacitated (Not Capacitated) and GSTO-inhibited and capacitated (GSTO Inhibited) mouse spermatozoa samples. (**B**) The intensity of the total protein tyrosine phosphorylation levels, shown in the Western blot at each time point for each treatment, were quantified using Image J and normalized to the respective intensity of alpha tubulin. Total tyrosine phosphorylation relative intensities were further normalized so that all time 0 values were 1. All measurements are the averages of three trials, all performed with different mice. Error bars represent standard error and * signifies statistical significance of *p* = 0.008 determined by a one-way ANOVA.

**Figure 5:**
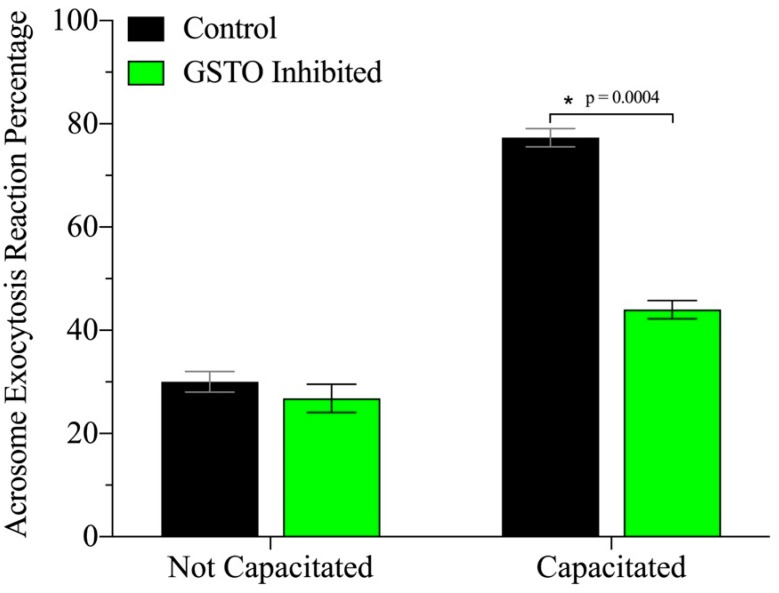
Sperm capacitation is reduced by the presence of the GSTO inhibitor when incubated in a capacitation medium. Sperm treatment was done through incubation for 25 min before in vitro capacitation was induced. The acrosome exocytosis reaction (a hallmark of capacitation) was induced using progesterone after 60 min of capacitation. Sperm samples were fixed and stained with PNA-647 and 4’,6-diamidino-2-phenylindole (DAPI) and scored based on acrosome labelling. At least 200 sperm per treatment were assessed per trial and grouped into acrosome intact or acrosome reacting/reacted. Statistical significance was determined by multiple t-tests and is denoted by *, *p* = 0.0004.

Inhibition of GSTO2’s catalytic site during in vitro capacitation in mice resulted in a dampening of the hallmark increase in tyrosine phosphorylation that occurs at the late stages of capacitation (Figure 4) without impairing sperm viability (Figure S3). These findings were also observed in boar spermatozoa (Figure S4). Most likely as a consequence of the diminished tyrosine phosphorylation events, mouse spermatozoa also demonstrated a significant decrease in their ability to successfully undergo acrosome exocytosis when GSTO enzymes were functionally inhibited prior to in vitro capacitation (Figure 5).

**Figure 6:**
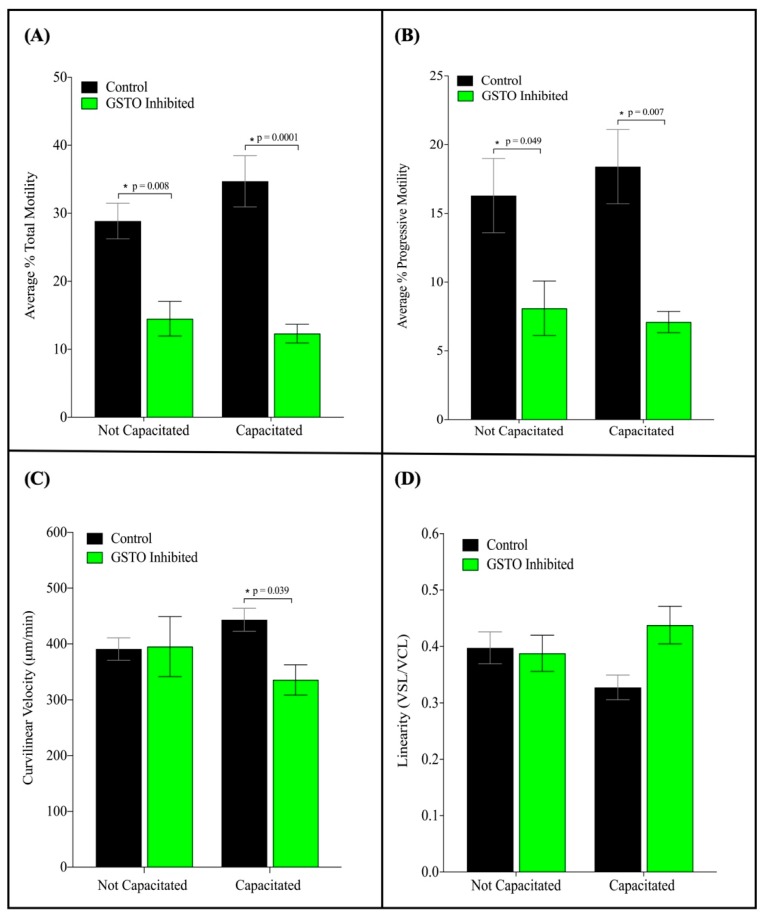
Computer-aided sperm analysis (CASA) of GSTO-inhibited and DMSO treated (Control) mouse spermatozoa after in vitro capacitation. Sperm motility parameters were analyzed on capacitated mouse spermatozoa that were either treated with DMSO (Control) or GSTO inhibitor prior to a 60-min incubation in capacitating medium at 37 degrees Celsius. The average total motility (**A**) was the combination of the progressive and non-progressive motility scores for each sample, whereas (**B**) shows solely the comparison of progressive motility. Both the total and progressive motility differences between the two treatment groups were statistically significant. The curvilinear velocity of GSTO inhibited sperm were also significantly decreased compared to controls when both treatment groups were capacitated (**C**). A higher linearity was also observed when spermatozoa were treated with the GSTO inhibitor (**D**), but the differences between the control and inhibited treatments were not found to be statistically significant. Error bars represent standard error. Statistical significance was determined using multiple t-tests and is denoted by *.

When GSTO inhibited spermatozoa were analyzed with computer-aided sperm analysis significant decreases in total and progressive motility were observed (Figure 6, Panel A and B). A significant decrease in the overall curvilinear velocity was also observed in capacitated samples (Figure 6, panel C). Additionally, while a trend of higher linearity, a measured ratio of straight line and curvilinear velocity, was observed in GSTO inhibited samples they were not found to be significant (Figure 6, Panel D). These findings suggest that the spermatozoa treated with the GSTO inhibitor were not as successful in reaching the ideal state of hyperactive activity when compared to the controls.

**Figure 7:**
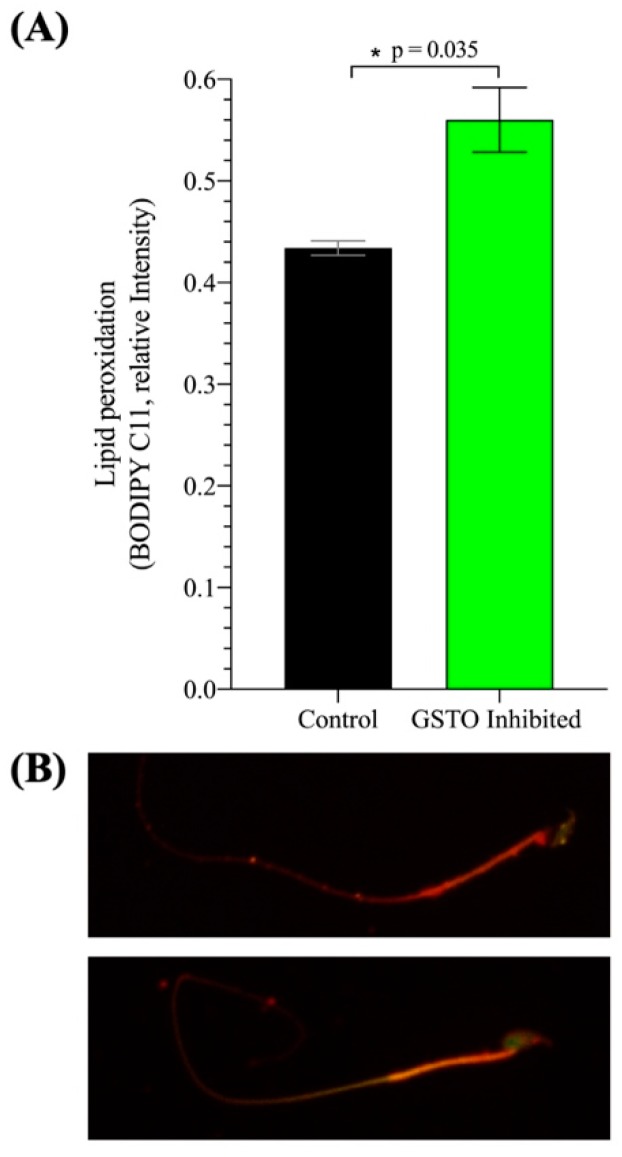
The assessment of sperm membrane lipid peroxidation after in vitro capacitation in mouse spermatozoa. (**A**) Lipid peroxidation levels were assessed based on the red and green fluorescence intensities of cells treated with the BODIPY 581/591 C11 probe. The relative intensity was calculated as the intensity of the green fluorescence over total fluorescence intensity. Spermatozoa were treated with the BODIPY 581/591 C11 probe and then incubated with either the GSTO Inhibitor or DMSO (Control) for 25 min before in vitro capacitation. After 60 min of in vitro capacitation, samples were fixed and imaged to determine the red and green fluorescence intensities of each cell. At least 200 cells were individually imaged in each treatment group for each trial, and a representative image from the control and GSTO inhibited groups is shown in (**B**). Three trials were performed, each with different mice, and error bars represent standard error. Statistical significance was determined using a t-test with Welch’s correction and is denoted by *.

**Figure 8:**
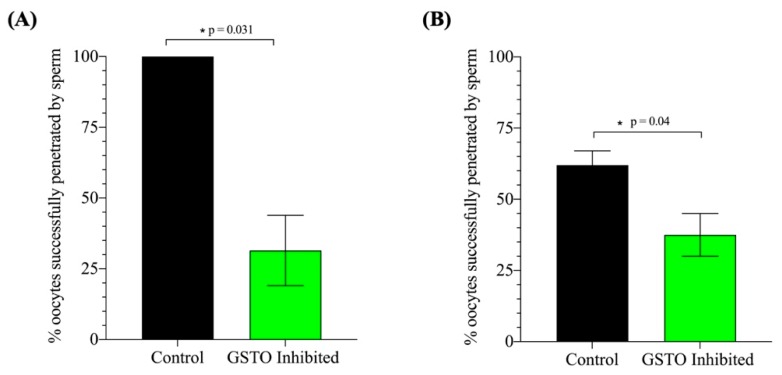
The assessment of sperm penetration during mouse and swine in vitro fertilization (IVF) using a low sperm concentration. Successful sperm penetration was assessed in both mouse (**A**) and swine (**B**) IVF models after 5–6 h of co-incubation in the fertilization droplet. Spermatozoa were pre-treated with either DMSO (Control) or GSTO inhibitor for 25 min before being placed in capacitation-inducing medium. Mouse sperm were incubated with cumulus–oophorus complexes at a concentration of 1 × 10^5^/mL and boar sperm was incubated with in vitro matured swine oocytes at a concentration of 1 × 10^4^/mL. Oocytes were washed and culture for 8 h (mouse) or 16 h (swine). Oocytes were fixed and stained with DAPI to assess pronuclear formation and sperm penetration. Statistical significance was assessed through a t-test with Welch’s correction. The data represent the adjusted average of three replicates and error bars represent standard error. Statistical significance is denoted by *.

Further investigations looked into the peroxidation of lipids within the plasma membrane of mouse spermatozoa after in vitro capacitation using the BODIPY 581/591 C11 probe that fluoresces red in a neutral state but is modified to a green fluorescence when lipids undergo peroxidation. The ratio of green fluorescence intensity over total fluorescence intensity was used as a measure lipid peroxidation and revealed a significant increase in lipid peroxidation when GSTO activity was inhibited (Figure 7). These findings were reinforced by a significant increase in the overall cellular reactive oxygen species levels of spermatozoa treated with the GSTO inhibitor compared to controls (Figure S5). Lastly, when in vitro fertilization was performed in both mouse and swine, there was a significant decrease in the sperm’s ability to successfully penetrate the oocyte (Figure 8).

## 4. Discussion

The present study demonstrates, for the first time, the presence of GSTO2 on the surface of spermatozoa and its participation in the regulation of ROS levels during capacitation. Surface protein biotinylation, indirect immunofluorescence with anti-GSTO2 antibodies, and a GSTO-specific fluorescent inhibitor revealed the surface localization of two GSTO2 isoforms covering the plasmalemma of both mouse and boar spermatozoa. The absence of GSTO2 reactivity on the surface of eutherian spermatids at the time of spermiation, and its presence on epididymal spermatozoa, suggests that the origin of the surface GSTO2 isoforms differs from the isoforms characterized within the perinuclear theca region of the sperm head [34].

After spermatogenesis, the spermatozoa released from the testis are fully formed but not adequately primed to interact with the oocyte. Transport in the epididymis allows sperm cells to mature and gain surface enzymes and molecules required to function optimally. Sperm maturation is driven exclusively by external factors within the luminal microenvironment of the epididymis and occurs in the absence of transcriptional and translational activity by the sperm [28,29,42,43,44,45,46]. Therefore, many of the proteins, enzymes, chaperones and cytokines that collectively contribute to sperm protection and function are acquired through the release of secretory products from the principal cells into the epididymal lumen [28,29,45,47,48]. Our immunohistochemical analysis of both mouse and boar epididymal segments suggests that GSTO2 is likely a constituent of these secretions. Since GSTO2 is considered a cytosolic protein that is non-glycosylated, it most likely finds its way into the epididymal lumen through the apocrine secretory pathway.

The caput epididymis is of critical importance in promoting sperm maturation and has been shown to have greater apocrine secretory activity than the epididymal regions it precedes [49]. Thus, the predominance of GSTO2 within the apical poles of the principal cells in the caput of both the mouse and boar epididymis is supportive of its role in promoting sperm maturation and enhancing fertilization capacity. The presence of two isoforms of GSTO2 on the sperm surface indicates a difference in their functional roles and/or temporal association with the sperm surface. The absence of the lower molecular weight isoform from the surface of boar spermatozoa after in vitro capacitation suggests that it may be removed as the membrane reorganizes in preparation for sperm–oocyte interactions. Without the ability to differentiate between the isoforms, their specific organizations on the sperm surface cannot be fully realized. Moreover, while this is the first report of GSTO2 on the surface of eutherian spermatozoa, we cannot exclude the possibility that our GSTO fluorescent inhibitor is also interacting with a GSTO1 isoform, since the inhibitor does not differentiate between members of the GST Omega family. However, neither we nor others have been successful in identifying GSTO1 in eutherian sperm.

The regulation of oxidative stress levels within capacitation is a true balancing act. Spermatozoa optimally function on the knife’s edge of beneficial versus detrimental oxidative stress levels and, therefore, must be equipped with the necessary antioxidant enzymes and substrates to effectively regulate their environment. When protective and antioxidant rich environmental barriers such as the seminal plasma are diluted within the female reproductive tract, spermatozoa must rely solely on membrane-bound, cytosolic, and mitochondrial antioxidant enzymes to maintain an effective oxidative equilibrium.

At low concentrations, reactive oxygen species (ROS) have been shown to positively affect capacitation and the acrosome exocytosis reaction and play central roles within most of the transduction pathways associated with the sperm acquiring its fertilizing ability [2,3,4,5,6,7,8,18]. Members of many of the oxidative-reductive regulating superfamilies’ have been characterized as sperm-resident proteins, such as thioredoxins, glutaredoxins, glutathione peroxidases (GPXs) glutathione-S-transferases (GSTs) and peroxiredoxins (PRDXs) [23,31,50,51,52,53,54,55]. Located internally or on the surface of the sperm plasma membrane, these enzymes function in diverse roles regulating transduction cascades, cell signaling, sperm–oocyte interactions and oxidative stress, facilitated by antioxidant molecules such as glutathione (GSH) and ascorbic acid (AA) [17,25,53,54,56].

Ascorbic acid (AA) or vitamin C is a potent single electron donor that acts as a scavenger of ROS in most organ systems [53,57]. AA is used in energetically favorable oxidation reactions to neutralize reactive and damaging compounds that contain an unpaired electron, such as hydroxide [53]. In both the germinal epithelium and the epididymis, AA levels are significantly higher than that of blood plasma, and a high concentration of AA within the seminal plasma has been shown to be positively correlated with sperm count, sperm motility and normal sperm morphology [26,27,53,58]. Previous reports have also shown that AA is required to protect sperm from endogenous ROS production at all stages of development and maturation in both the germinal layer and the epididymis [59,60]. Therefore, its replenishment within epididymal transport and capacitation processes may prove vital in maintaining the oxidative-reductive homeostasis of sperm as they prepare for fertilization. The recycling of dehydroascorbate (DHA) into its reduced ascorbate state can be accomplished through various mechanisms, including the direct reduction by glutathione and the enzymatic reduction by various thiol transferases and NADPH-dependent reductases [61]. One such enzyme that can facilitate this reaction is glutathione-s-transferase omega 2 (GSTO2). Past reports have found that GSTO2 has the highest dehydroascorbate reductase functionality within mammalian systems, with a higher affinity for DHA than glutathione itself [30,37,62]. Therefore, in sperm maturation and capacitation, two highly oxidative processes that utilize AA as a major source of reducing power, the presence of a high affinity DHA recycling enzyme may be critical in maintaining the required concentration of AA to effectively defend against damaging levels of oxidative stress.

The sperm plasma membrane is a rich lipid bilayer that is highly susceptible to lipid peroxidation by oxidative stress [10,11,12,63]. Damage to the membrane structure has previously been shown to have wide reaching functional implications in sperm, as membrane fluidity and surface proteins have large roles in both capacitation and sperm–oocyte interactions [9,13,14,15,16,17]. Our results show that the absence of GSTO2’s catalytic activity negatively impacts the spermatozoon’s ability to prevent lipid peroxidation within the plasmalemma during capacitation, resulting in the impairment or dysregulation of membrane-associated processes such as the acrosome exocytosis reaction, sperm motility and, ultimately, the sperm’s ability to penetrate the oocyte. Therefore, while GSTO2 may not have a direct role within each of these processes, it is possible that its functional involvement in the regulation of the membranes oxidative state may have indirect implications on the sperm’s overall fitness.

Limitations of this study were that our functional inhibitor was permeable and did not discriminate between different GSTO enzymes and isoforms. However, to date, only GSTO2 has been shown to reside on or within spermatozoa. We recently reported that GSTO2 isoforms within the post-acrosomal and perforatorial regions of the perinuclear theca (PT) facilitate the post-fertilization nuclear decondensation and male nuclear transition [50]. For this reason, we did not investigate any events beyond sperm penetration where the PT isoforms would functionally participate. That these PT-resident GSTO2s could be involved in the capacitation process is unlikely, as they are firmly anchored to the PT and require harsh solubilization agents such as 1M NaOH to release them from the PT [34]. Due to this insolubility, their catalytic sites could be masked until solubilization in the oocyte cytoplasm during fertilization. Even if they were involved, their coverage compared to the surface GSTO2 is limited, and thus their functional contribution would be expected to be proportionally small.

## 5. Conclusions

Our investigations identify GSTO2 isoforms as functionally active surface-borne enzymes on mouse and boar spermatozoa. We propose that GSTO2 may facilitate the regulation of the oxidative environment of the plasmalemma indirectly, through the replenishment of antioxidant molecules such as ascorbic acid and glutathione. Overall, this study highlights the importance of oxidative-reductive regulation and demonstrates the wide-reaching negative implications its dysregulation can have on overall sperm fitness.

## Supplementary Materials

The following are available online at https://www.mdpi.com/2076-3921/8/12/601/s1, Figure S1: Mouse and boar GSTO sequence comparison, Figure S2: GSTO inhibitor binding specificity in whole mouse and boar spermatozoa, Figure S3: The Assessment of sperm viability between treatment groups, Figure S4: The level of protein tyrosine phosphorylation during in vitro capacitation in boar spermatozoa, Figure S5: The difference in cellular reactive oxygen species (ROS) levels of GSTO Inhibited and Control capacitated spermatozoa.
