# Supplementary material for: GSTO2 Isoforms Participate in the Oxidative Regulation of the Plasmalemma in Eutherian Spermatozoa during Capacitation
Source: Antioxidants (Basel). 2019 Nov 29;8(12):601. doi: 10.3390/antiox8120601 (PMC6943649; doi:10.3390/antiox8120601)
Supplement: Supplementary file 1 [file antioxidants-08-00601-s001.zip › Antioxidants 631334 Supplementary Figures/Supplementary Figure 4.pdf]

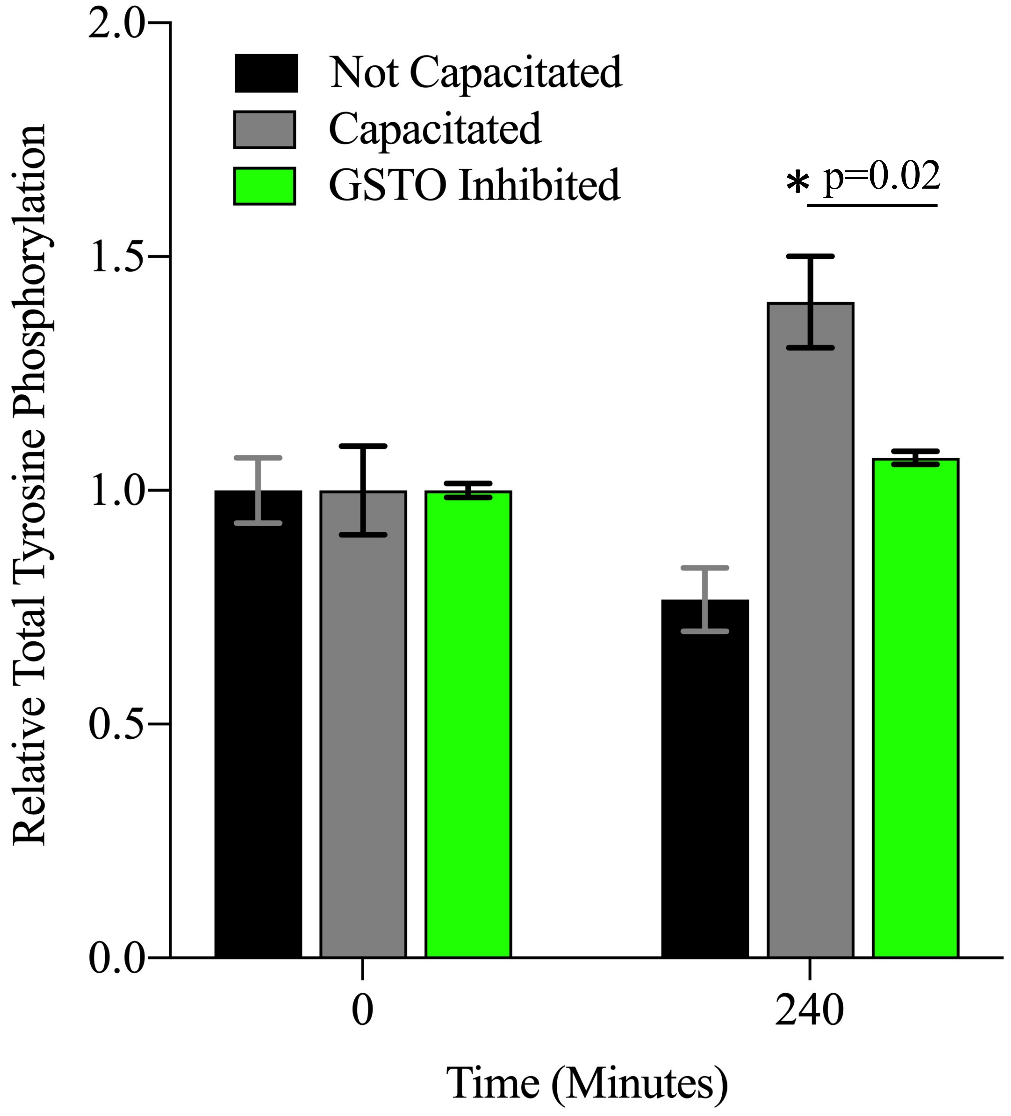


**Supplementary Figure 4. The level of protein tyrosine phosphorylation during *in vitro* capacitation in boar spermatozoa.** Samples were taken every 80 minutes during induced *in vitro* capacitation. Both capacitated and negative control (Not Capacitated) samples were kept in the same conditions to standardize all treatment groups. Total protein tyrosine phosphorylation levels were quantified using Image J and standardized through anti-alpha tubulin reactivity (Sigma, T6074). Tyrosine phosphorylation intensity measurements were adjusted so all time 0 measurements were an intensity of 1 to simplify the comparison. All measurements are the averages of three trials. Error bars represent standard error and * signifies statistical different of p=0.02.
