# Supplementary material for: GSTO2 Isoforms Participate in the Oxidative Regulation of the Plasmalemma in Eutherian Spermatozoa during Capacitation
Source: Antioxidants (Basel). 2019 Nov 29;8(12):601. doi: 10.3390/antiox8120601 (PMC6943649; doi:10.3390/antiox8120601)
Supplement: Supplementary file 1 [file antioxidants-08-00601-s001.zip › Antioxidants 631334 Supplementary Figures/Supplementary Figure 5.pdf]

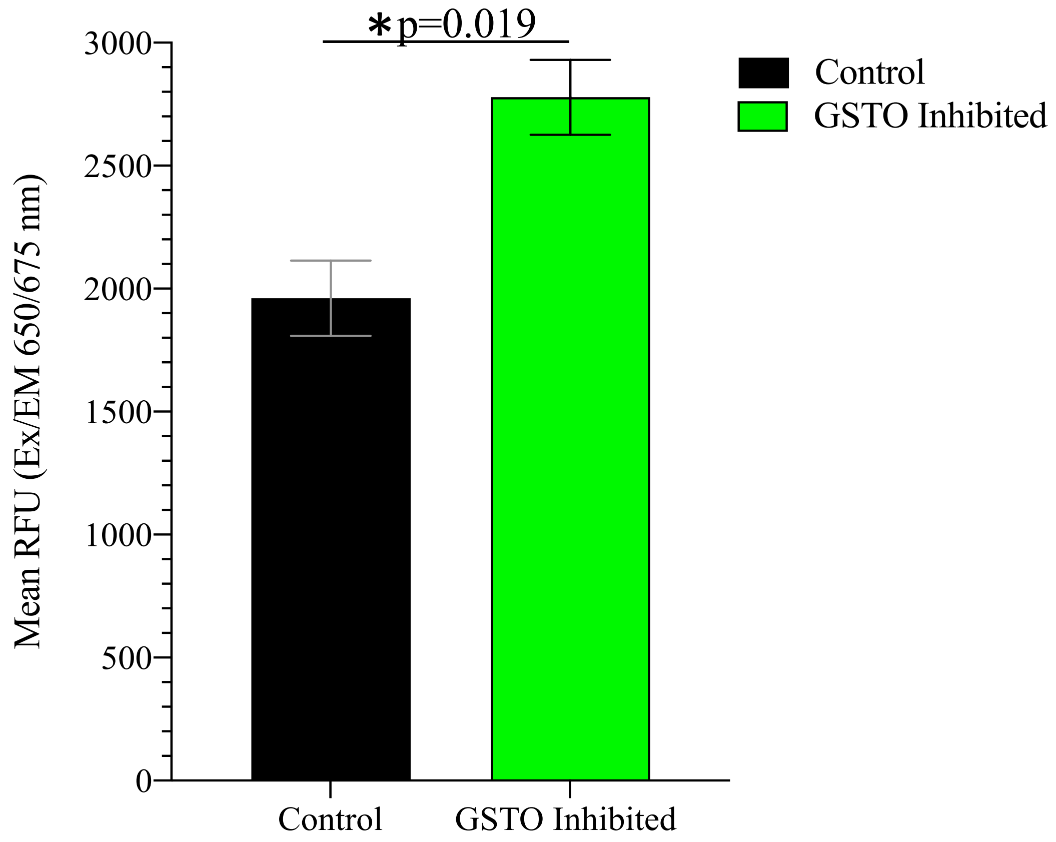

Supplementary Figure 5. The level of cellular ROS levels in mouse spermatozoa after *in vitro* capacitation. Mouse spermatozoa were treated with DMSO (Control) or the GSTO inhibitor for 25 minutes before induced *in vitro* capacitation. Samples were then processed using the Cellular ROS Detection Kit (ab186029) and analyzed using flow cytometry. The mean relative fluorescence units (RFU) for each treatment is shown and represent the average mean intensity from three trials using three different mice and measuring at least 10,000 cells for each treatment per trial. The error bars represent standard error and * signifies a statistical significance of p=0.019.
