# Supplementary material for: GSTO2 Isoforms Participate in the Oxidative Regulation of the Plasmalemma in Eutherian Spermatozoa during Capacitation
Source: Antioxidants (Basel). 2019 Nov 29;8(12):601. doi: 10.3390/antiox8120601 (PMC6943649; doi:10.3390/antiox8120601)
Supplement: Supplementary file 1 [file antioxidants-08-00601-s001.zip › Antioxidants 631334 Supplementary Figures/Supplementary Figure 2.pdf]

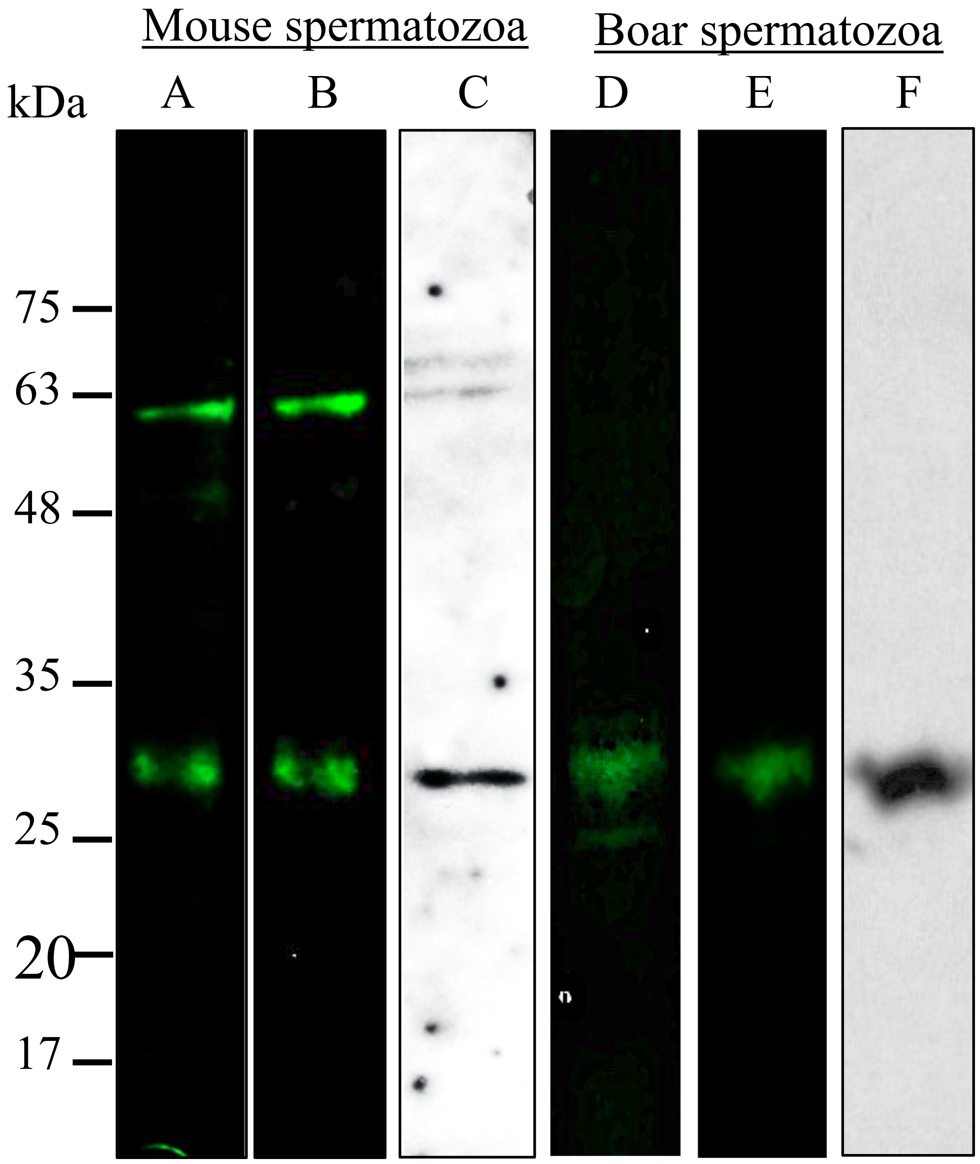

Supplementary Figure 2. GSTO inhibitor binding specificity in whole mouse and boar spermatozoa.

Mouse and boar whole sperm incubated with a GSTO specific fluorescent probe (C7025). Pre-incubated mouse whole sperm with the GSTO inhibitor shows binding to two proteins, at approximately 28kDa and 56 kDa. Whereas the GSTO inhibitor binds at approximately 32 kDa in boar whole sperm. These molecular masses correspond to the reported molecular masses of GSTO2 and its functional dimer. The bands are shown through a fluorescence gel detection of the bound inhibitor (Mouse (**A**) and Boar (**D**)), as well as on the PVDF membrane (Mouse (**B**) and Boar (**E**)). Western blot analysis of the same membrane using a GSTO2 specific antibody shows direct correlation with the fluorescent inhibitor binding specificity in both species (Mouse (**C**) and Boar (**F**)).
