# Supplementary material for: GSTO2 Isoforms Participate in the Oxidative Regulation of the Plasmalemma in Eutherian Spermatozoa during Capacitation
Source: Antioxidants (Basel). 2019 Nov 29;8(12):601. doi: 10.3390/antiox8120601 (PMC6943649; doi:10.3390/antiox8120601)
Supplement: Supplementary file 1 [file antioxidants-08-00601-s001.zip › Antioxidants 631334 Supplementary Figures/Supplementary Figure 3.pdf]

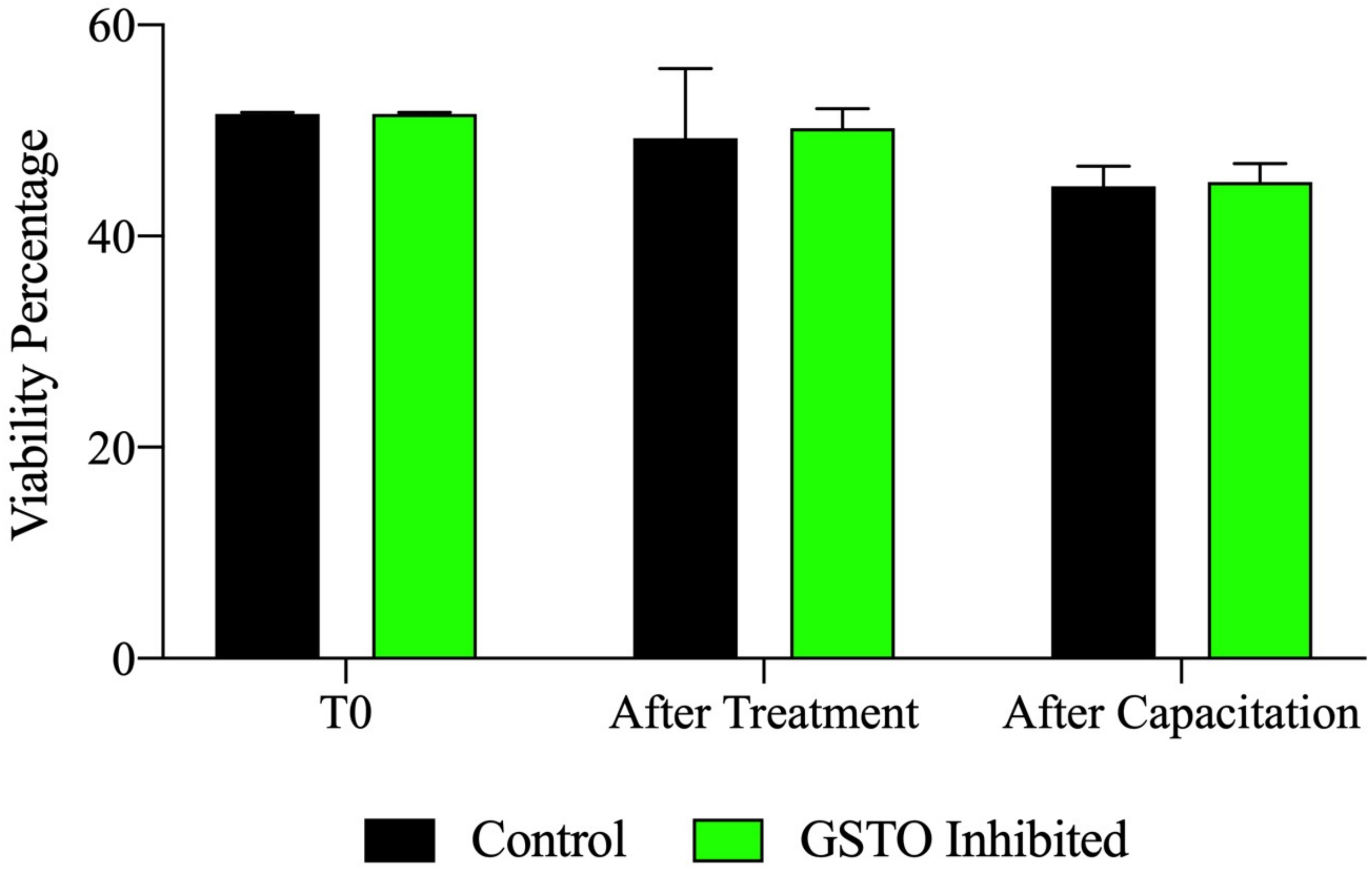

Supplementary Figure 3. The assessment of sperm viability between treatment groups. Mouse spermatozoa from C57BL/6 males were treated with either the GSTO Inhibitor or no treatment for 25 minutes prior to *in vitro* capacitation and samples were taken prior to treatment (T0), after treatment and after 90 minutes of *in vitro* capacitation (After Capacitation). Samples were fixed and mounted onto glass slides and stained with an Eosin-Nigrosin staining procedure that shows membrane integrity. At least 200 cells were examined from each treatment and cells were scored as either dead or alive based on if they stained white or light pink (viable) or dark red (not-viable). Three trials were performed all with different mice and the data is presented as the average of all trials. Error bars represent standard error. There was no significant difference in viability between any of the treatment groups.
