# Supplementary material for: GSTO2 Isoforms Participate in the Oxidative Regulation of the Plasmalemma in Eutherian Spermatozoa during Capacitation
Source: Antioxidants (Basel). 2019 Nov 29;8(12):601. doi: 10.3390/antiox8120601 (PMC6943649; doi:10.3390/antiox8120601)
Supplement: Supplementary file 1 [file antioxidants-08-00601-s001.zip › Antioxidants 631334 Supplementary Figures/Supplementary Figure 1.pdf]

```
mGSTO2      MSGDLSRCLGKGSCPPGPVPEGVIRIYSMRFCPYSHRARLVLKAKGIRHEVININLKSKP
bGSTO2      MTDDATRTLGKGSIPPGPVPEGVIRLYSMRFCPYAHRTRLVLRAKGIRHEVININLRNKP
mGSTO1      MSGESSRSLGKGSAPPGPVPEGQIRVYSMRFCPFAQRTLMVLKAKGIRHEVININLKNKP
bGSTO1      MSGGSARSLGKGSAPPGPVPEGLIRVYSMRFCPFAQRTLLVLNAKGIRHQVININLKNKP
            *:.  :* ***** ******** **:*******:::*: :**.******:******:.**

mGSTO2      DWYYTKHPFGQIPVLENSQCQLVYESVIACEYLDDVYPGRKLFPYDPYERARQKMLLELF
bGSTO2      EWYFTKHPFGQIPVLENSKCQLIYESVIACEYLDDAYPGRKLYPYDPYERARQKMLLELF
mGSTO1      EWFFEKNPLGLVPVLENSQGHLVTESVITCEYLDEAYPEKKLFPDDPYKKARQKMTLESF
bGSTO1      EWFFQKNPSGLVPVLENSQGQLIYESAITCEYLDEAYPGKKLLPDDPYEKACQKMVFELS
            :*:: *:* * :******: :*: **.*:*****:.** :** * ***::* *** :*

mGSTO2      CKVPPLSKECLIALRCGRDCTDLKVALRQELCNMEEILEYQNTTFFGGDCISMIDYLVWP
bGSTO2      YKVPHLTKECLVALRCGRDCGDLKLALRQEFCNLEEILGYQNTVFFGGDCISMIDYLFWP
mGSTO1      SKVPPLIASF-VRSKRKEDSPNLREALENEFKKLEEGMDN-YKSFLGGDSPSMVDYLTWP
bGSTO1      SKVPPLLIRF-IRRENEADCSGLKEELRKEFSKLEEVLTKKKTTYFGGSSLSMIDYLIWP
             *** *     :  .   *. .*:  *.:*: ::** :    . ::**.. **:*** **

mGSTO2      WFERLDVYGLADCVNHTPMLRLWIASMKQDPAVCALHTDKSVFLGFLNLYFQNNPCAFDF
bGSTO2      WFERLEVYGIADCVNHTPALRLWIAAMKQDPTVCSLLTDKNTFLGFLNLYFQNNPGAFDY
mGSTO1      WFQRLEALELKECLAHTPKLKLWMAAMQQDPVASSHKIDAKTYREYLNLYLQDSPEACDY
bGSTO1      WFERLEALELNECIDHTPKLKLWMAAMMKDPAVSALHIEPRDLRAFNDLYLQNSPEACDY
            **:**:.  : :*: *** *:**:*:* :**...:   :      : :**:*:.* * *:

mGSTO2      GLCNPIIR    248
bGSTO2      GLSC----    244
mGSTO1      GL------    240
bGSTO1      GL------    241
            **
```

Supplementary Figure 1. Mouse and Boar GSTO sequence comparison. The mouse glutathione-s-transferase omega 2 (mGSTO2), boar glutathione-s-transferase omega 2 (bGSTO2), mouse glutathione-s-transferase omega 1 (mGSTO1) and boar glutathione-s-transferase omega 1 (bGSTO1) protein sequence alignment. The active site cysteine (aa32) is identified by a red arrow and the inhibitor (CellTracker C7025) binding site in each sequence is identified by a green underline. Fully conserved residues within the sequences are indicated by an asterisk (*), residues with strong similarity are indicated by a colon (:) and weakly similar residues are indicated by a period (.). The inhibitor is a membrane permeable probe that covalently binds to the active site and fluoresces when bound.
